# Exploration of the role of oxidative stress-related genes in LPS-induced acute lung injury via bioinformatics and experimental studies

**DOI:** 10.1038/s41598-023-49165-3

**Published:** 2023-12-09

**Authors:** Yuanshui Liu, Huamei Li, Yanhong Ouyang, Yan Zhang, Pinhua Pan

**Affiliations:** 1 Department of Emergency Medicine, Hainan General Hospital, Hainan Affiliated Hospital of Hainan Medical University, Haikou, 570311 People’s Republic of China; 2 Department of Ultrasound, Hainan General Hospital, Hainan Affiliated Hospital of Hainan Medical University, Haikou, 570311 People’s Republic of China; 3 Department of Respiratory Medicine, Key Cite of National Clinical Research Center for Respiratory Disease, Xiangya Hospital, Central South University, Changsha, 410008 People’s Republic of China

**Keywords:** Bioinformatics, Genomic analysis

## Abstract

During the progression of acute lung injury (ALI), oxidative stress and inflammatory responses always promote each other. The datasets analyzed in this research were acquired from the Gene Expression Omnibus (GEO) database. The Weighted Gene Co-expression Network Analysis (WGCNA) and limma package were used to obtain the ALI-related genes (ALIRGs) and differentially expressed genes (DEGs), respectively. In total, two biological markers (Gch1 and Tnfaip3) related to oxidative stress were identified by machine learning algorithms, Receiver Operator Characteristic (ROC), and differential expression analyses. The area under the curve (AUC) value of biological markers was greater than 0.9, indicating an excellent power to distinguish between ALI and control groups. Moreover, 15 differential immune cells were selected between the ALI and control samples, and they were correlated to biological markers. The transcription factor (TF)-microRNA (miRNA)-Target network was constructed to explore the potential regulatory mechanisms. Finally, based on the quantitative reverse transcription polymerase chain reaction (qRT-PCR), the expression of Gch1 and Tnfaip3 was significantly higher in ALI lung tissue than in healthy controls. In conclusion, the differences in expression profiles between ALI and normal controls were found, and two biological markers were identified, providing a research basis for further understanding the pathogenesis of ALI.

## Introduction

Acute respiratory distress syndrome (ARDS) is a heterogeneous syndrome of acute lung injury (ALI) in humans with multiple etiologies^1^, which can be caused by direct lung injury from pneumonia and aspiration or indirect injury from non-pulmonary sepsis and pancreatitis^2^. ARDS is mainly manifested by direct or indirect alveolar-capillary damage^3^. Cell death, loss of cell–cell junctions and/or cell–matrix attachment leads to epithelial (direct ARDS) or endothelial (indirect ARDS) damage and barrier dysfunction^4^, characterized by pulmonary capillary congestion, interstitial edema, alveolar edema, alveolar collapse, alveolar hemorrhage and hyaline membrane formation, resulting in non-cardiogenic pulmonary edema and hypoxic respiratory failure^5,6^. The mortality rate of ARDS continues to be maintained at a high level of approximately 40% due to the lack of effective drug therapy^1^.

Oxidative stress is the result of a disruption in the balance between reactive oxygen species (ROS) and antioxidant defense systems, and the resulting cellular damage is strongly associated with the occurrence and development of many human diseases^7^. Excessive production of ROS causes oxidative damage to molecules and cells under various pathological conditions, including ALI/ARDS, which also upregulates multiple inflammatory cytokines and tends to maintain a vicious malignancy of damage by recruiting more inflammatory cells, which ultimately leading to severe lung tissue damage^8^. Previous findings have shown that destructive oxidants produced by oxidative stress can directly damage tissues during the acute exudative phase of ALI/ARDS^9^. Also through interaction with the inflammatory response, some oxidants (e.g., ROS) act as inflammatory signaling molecules to activate NF-κB, NLRP3 and other inflammatory pathways, exacerbating ALI/ARDS^10^. However, the mechanisms regulating oxidative stress in ALI are not fully elucidated.

Lipopolysaccharide (LPS) is a specific composition of the Gram negative bacterial cell wall, which is one of the main drivers of ALI/ARDS^11^. It can indirectly damage alveolar epithelial cells by activating the release of inflammatory mediators from alveolar macrophages, neutrophils and other immune cells. Furthermore, it directly acts on TLR4 on alveolar epithelial cells, causing direct oxidative stress and inflammatory response in alveolar epithelial cells, leading to breakage of tight junctions between alveolar epithelial cells, increase in cell gaps and increased permeability^12^.

**Figure 1:**
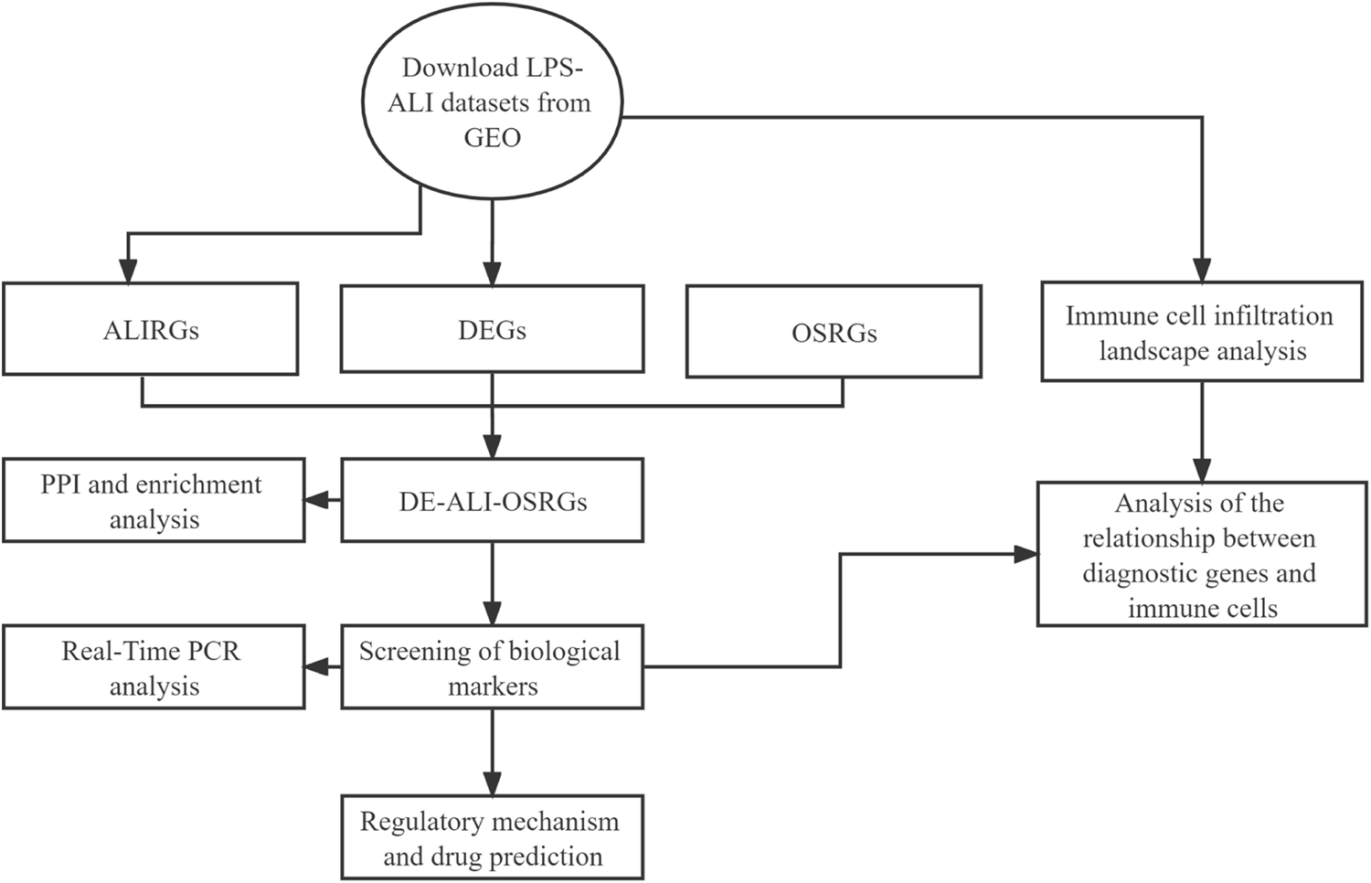
Logical flow of the analysis. ALIRGs ALI-related genes, DEGs differentially expressed genes, OSRGs oxidative stress-related genes, DE-ALI-OSRGs differentially expressed OSRGs in ALI.

In this study, we performed a series of bioinformatics analyses and basic experiments based on transcriptional profiling data of ALI mice samples and normal control mice lung tissues from the GEO database, aiming to analyze the mechanism of action of oxidative stress-related genes (OSRGs) in LPS-induced lung injury and the relationship with immune cell infiltration. Finally, we obtained the core genes of oxidative stress closely related to LPS-induced ALI, which were used to predict the drugs that may play a therapeutic role and provide a research basis for the study of the pathogenesis and treatment of ALI. The process of data analysis is illustrated in Fig. 1.

## Results

### Selection of 152 ALI-related genes (ALIRGs) by weighted gene co-expression network analysis (WGCNA)

**Figure 2:**
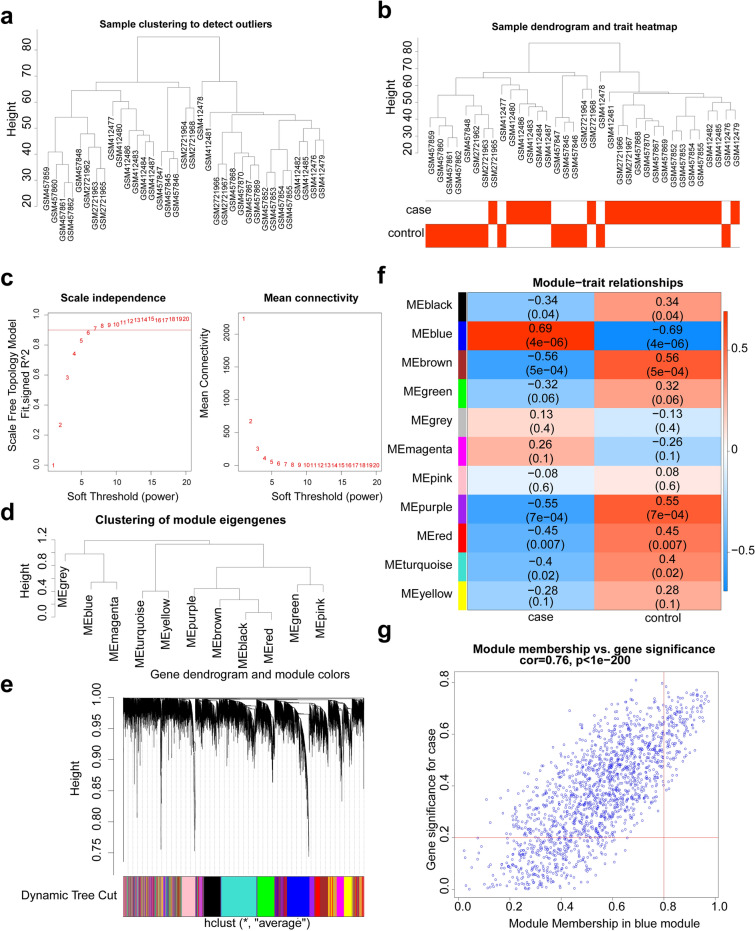
Weighted gene co-expression network analysis (WGCNA). (**a**) The clustering of samples in the merged dataset to remove outlier. (**b**) Clustering of merged data samples and phenotype information. (**c**) The determination of soft threshold. Seven was determined as the optimal soft threshold. (**d**) The clustering of module eigengenes. (**e**) Identification of gene co-expression modules. (**f**) Heatmap of correlation between modules and clinical traits. (**g**) The module membership (MM) and gene significance (GS scatter) plots of blue module.

The samples of the GSE16409, GSE18341 and GSE102016 datasets were discretely distributed before merging, and the sample data (ALI = 21 and control = 14) was uniform after batch processing (Supplementary Fig. 1a,b). To identify the ALIRGs, the WGCNA was performed in the combined dataset. As shown in the Fig. 2a,b, the cluster of samples was performed well with no outlier samples. The soft threshold was equal to 7 when the ordinate R^2^ reached the threshold of 0.85 (red line). Simultaneously, the network was closer to a scale-free distribution, and the mean connectivity also close to 0. Thus, optimal soft threshold was selected as 7 (Fig. 2c). In total, 11 candidate modules were selected to obtain key module (Fig. 2d,e). The blue module had the highest correlation with ALI samples, which contained 1642 genes (Fig. 2f). Among 1642 genes, 152 genes were selected as ALIRGs with |Modulemembership (MM)| > 0.8 and |Genesignificance (GS)| > 0.2 (Fig. 2g).

### Identification of 17 differentially expressed OSRGs in ALI (DE-ALI-OSRGs)

**Figure 3:**
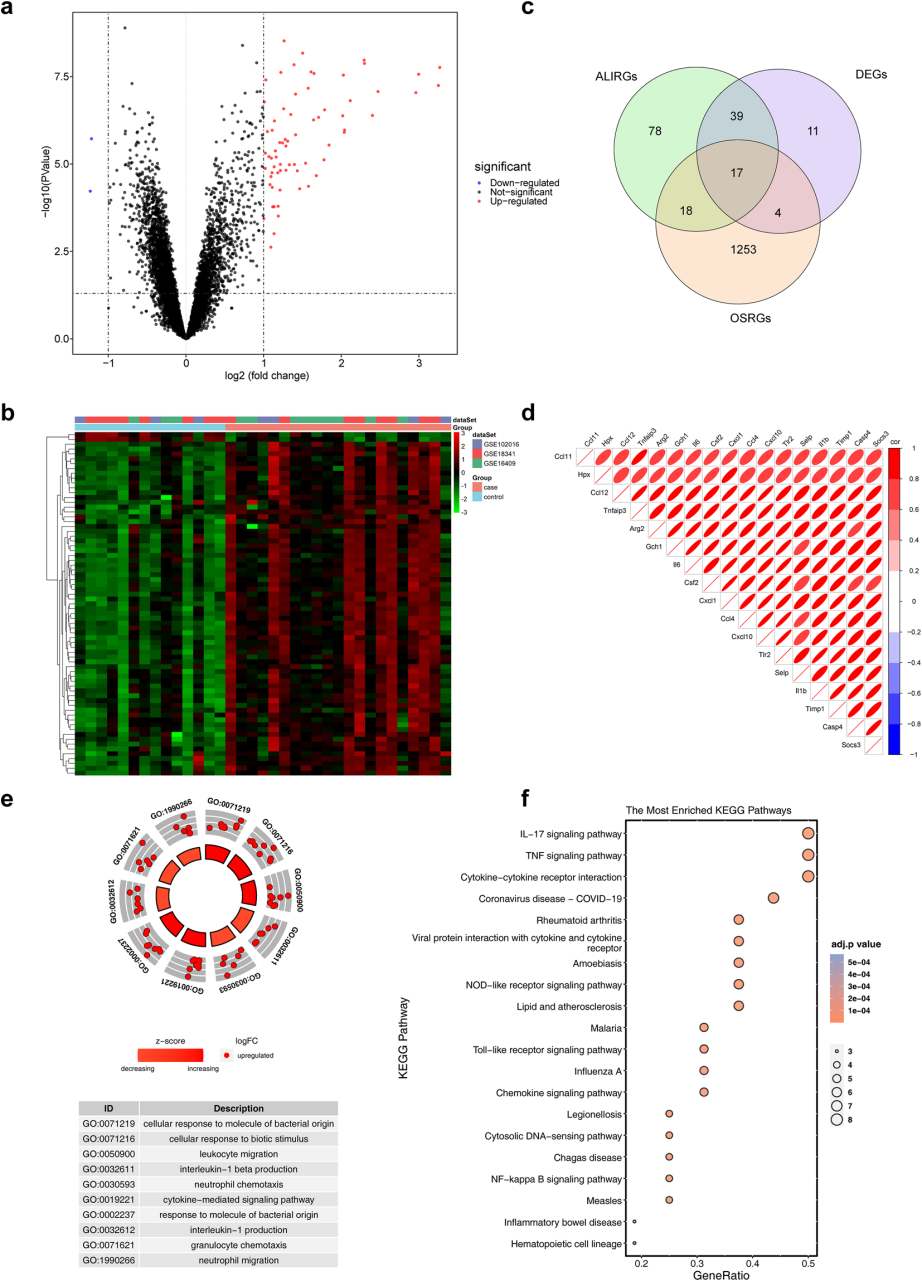
Identification of differentially expressed OSRGs in ALI (DE-ALI-OSRGs) and functional enrichment analysis. (**a**) The volcano map of differentially expressed genes (DEGs) between ALI and control samples. (**b**) The heat map of DEGs between ALI and control samples. (**c**) Venn diagram for certification of DE-ALI-OSRGs. (**d**) The correlation between DE-ALI-OSRGs. The shadow of the ellipse in each color box represents the size of the correlation between two genes. The greater the correlation, the narrower the ellipse. The smaller the correlation, the rounder the ellipse. Red represents a positive correlation and blue represents a negative correlation. (**e**) The gossip chart of top10 Gene Ontology (GO) terms enriched by DE-ALI-OSRGs. Z-score is an value which give a hint if the biological process (/molecularfunction/cellular components) is more likely to be decreased or increased. LogFC is used to represent the number of up- and down-regulated genes. (**f**) The bubble chart of top20 Kyoto Encyclopedia of Genes and Genomes (KEGG) pathways enriched by DE-ALI-OSRGs.

A total of 71 differentially expressed genes (DEGs) were selected with |log_2_FC| > 1 and *p* < 0.05 (Fig. 3a). Among them, 69 genes were up-regulated and 2 genes were down-regulated in the ALI samples. Heat map of the expression of up- and down-regulated genes in the ALI and control groups in the combined dataset was plotted (Fig. 3b). Finally, 17 DE-ALI-OSRGs were obtained by overlapping the ALIRGs, DEGs, and OSRGs (Fig. 3c). There were strong correlations among 17 DE-ALI-OSRGs (Fig. 3d). In order to investigate the potential molecular mechanisms of DE-ALI-OSRGs, Gene Ontology (GO) and Kyoto Encyclopedia of Genes and Genomes (KEGG) enrichment analyses were implemented^13,14^. A total of 946 GO terms were enriched with adj.p < 0.05 and count > 2, including 903 BP terms and 43 MF terms. The top10 GO terms were shown in Fig. 3e, such as cellular response to molecule of bacterial origin, cellular response to biotic stimulus and leukocyte migration. Based on the z-score and logFC, these GO terms were significantly enriched by up-regulated, and they were more likely to be increased. Meanwhile, 42 KEGG pathways were enriched with adj.p < 0.05 and count > 2. The top 20 KEGG pathways were showed in the Fig. 3f, such as IL-17 signaling pathway, TNF signaling pathway, and cytokine-cytokine receptor interaction.

### Gch1 and Tnfaip3 were screened as biological markers

**Figure 4:**
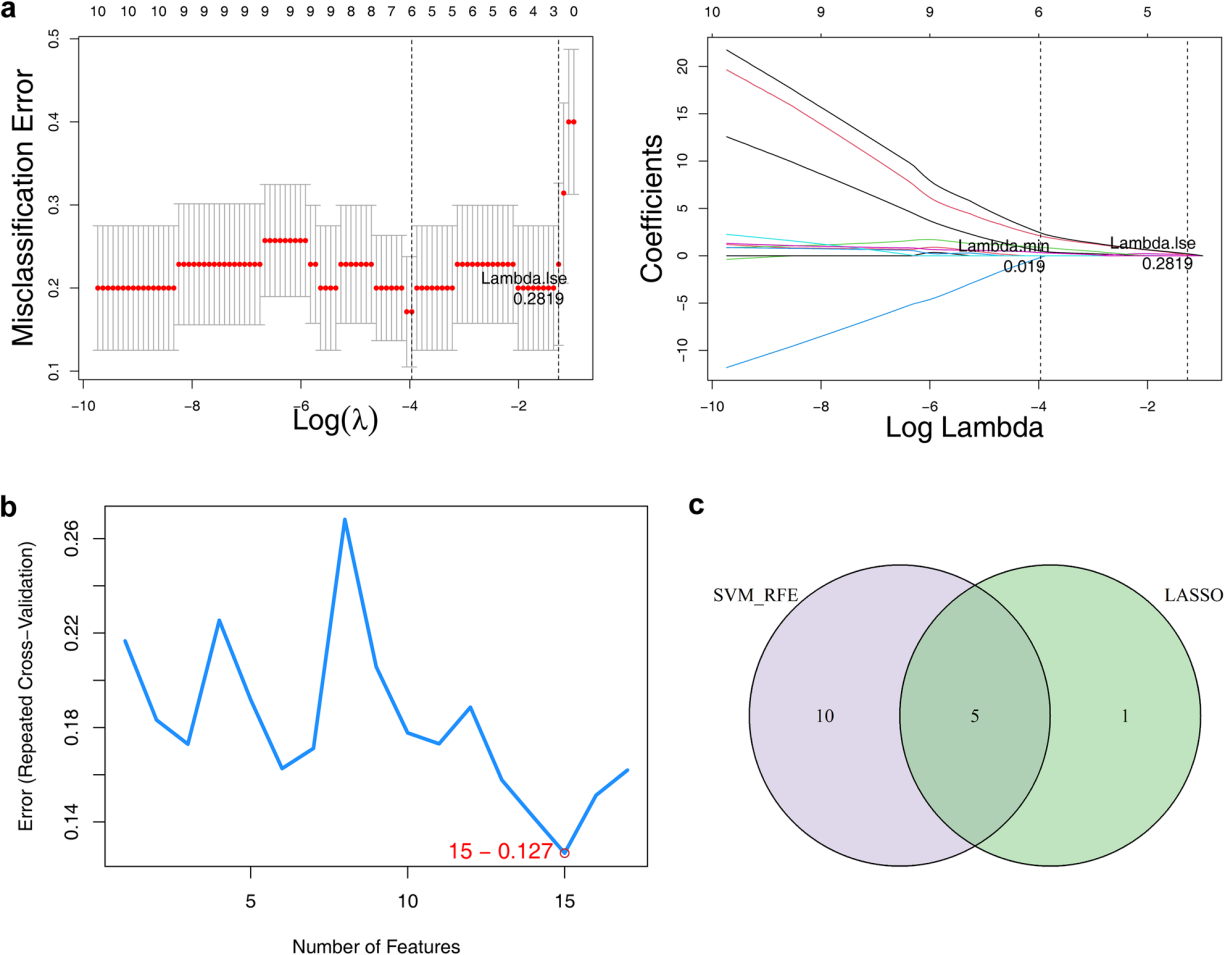
Identification of candidate biological markers. (**a**) Logic factor penalty plot and cross validation error curve of Least-Absolute Shrinkage and Selection Operator (LASSO) model. Each curve represents the trajectory of each independent variable coefficient. (**b**) The accuracy of support vector machine recursive feature elimination (SVM-RFE) model. (**c**) Wayne chart of characteristic genes identified by LASSO and SVM-RFE.

To screen ALI candidate biological markers, the Least-Absolute Shrinkage and Selection Operator (LASSO) and support vector machine recursive feature elimination (SVM-RFE) models were performed in the combined dataset based on the DE-ALI-OSRGs. Then, 6 candidate biological markers were selected with the lambda.min = 0.019 by LASSO, including Arg2, Ccl4, Gch1, Hpx, Socs3, and Tnfaip3 (Fig. 4a). Moreover, 15 candidate genes were selected by SVM-RFE based on gene importance ranking and error rate (Fig. 4b, Table S1). Finally, the 5 overlapping genes were selected from the results of LASSO and SVM-RFE as candidate biological markers (Fig. 4c), including Arg2, Ccl4, Gch1, Hpx and Tnfaip3.

**Figure 5:**
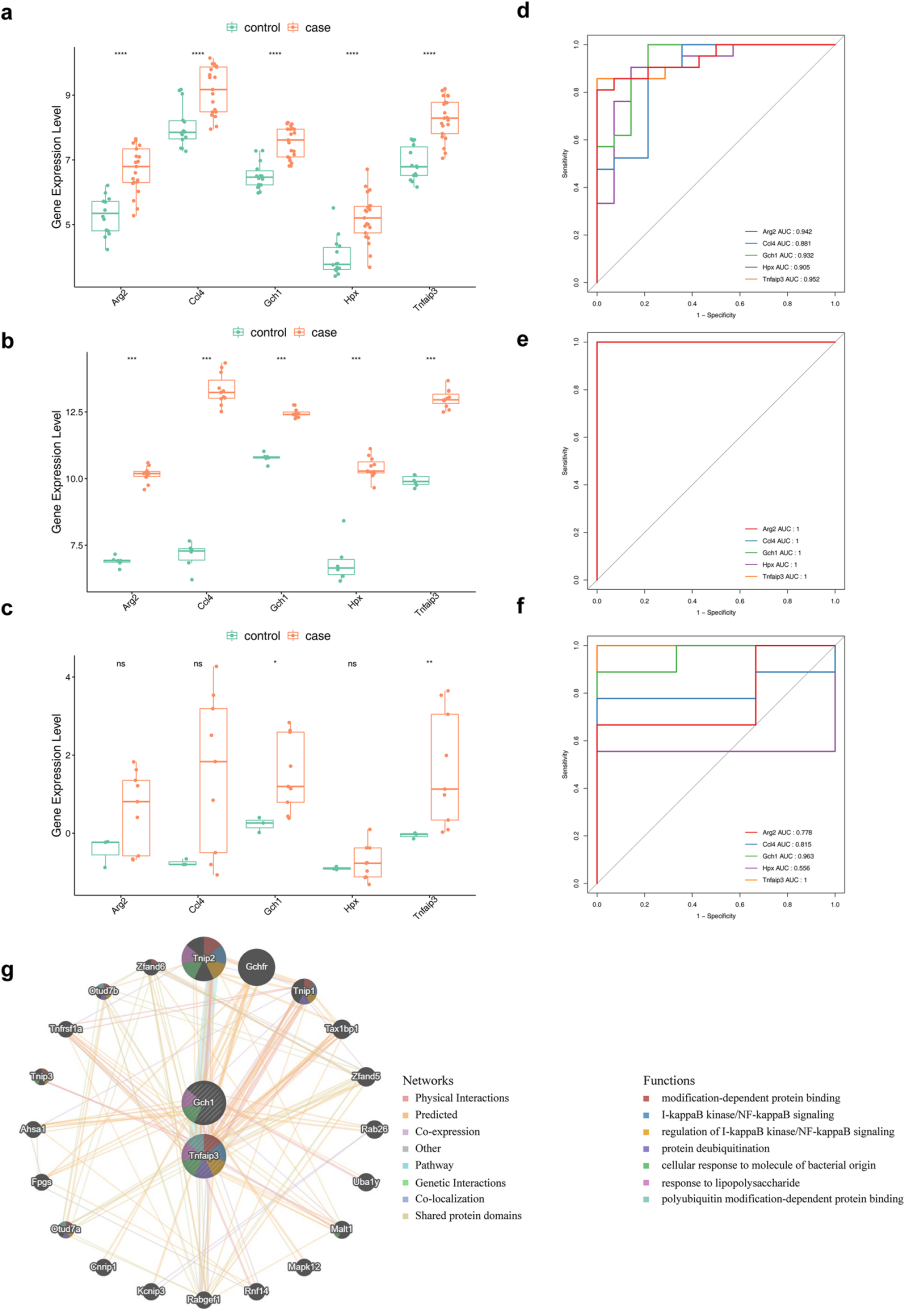
Identification of biological markers. (**a**) The expression levels of key genes in the merged dataset, ****p value < 0.0001. (**b**) The expression levels of key genes in the GSE104214 dataset, ***p value < 0.001. (**c**) The expression levels of key genes in the GSE17355 dataset, *ns* not significant, *p value < 0.05, **p value < 0.01. (**d**) Receiver Operator Characteristic (ROC) curves of key genes in the merged dataset. *AUC* area under the curve. (**e**) ROC curves of key genes in the GSE104214 dataset. (**f**) ROC curves of key genes in the GSE17355 dataset. (**g**) The protein–protein interaction (PPI) network of biological markers.

To further excavate the biological markers, expression analysis was performed and ROC curve was painted in the combined dataset, GSE104214 dataset, and GSE17355 dataset, respectively. Figure 5a–c demonstrated the expression of Gch1 and Tnfaip3 was significantly different between ALI and control samples, and the expression was higher in ALI samples in all three datasets. In addition, the AUC value of 4 genes (Arg2, Ccl4, Gch1 and Tnfaip3) was all greater than 0.7, indicating a decent ability to distinguish between ALI and control samples (Fig. 5d–f). In summary, Gch1 and Tnfaip3 with differential expression and AUC values greater than 0.9 were treated as biological markers for subsequent analysis. To explore the interaction among two biological markers, the protein- protein interaction (PPI) network was constructed. As shown in Fig. 5g, Tnip2, Gchfr, and Tnip1 had stronger interaction with biological markers. Additionally, both Gch1 and Tnfaip3 were associated with cellular response to molecule of bacterial origin and response to lipopolysaccharide.

### Gene set enrichment analysis (GSEA) enrichment analysis

**Figure 6:**
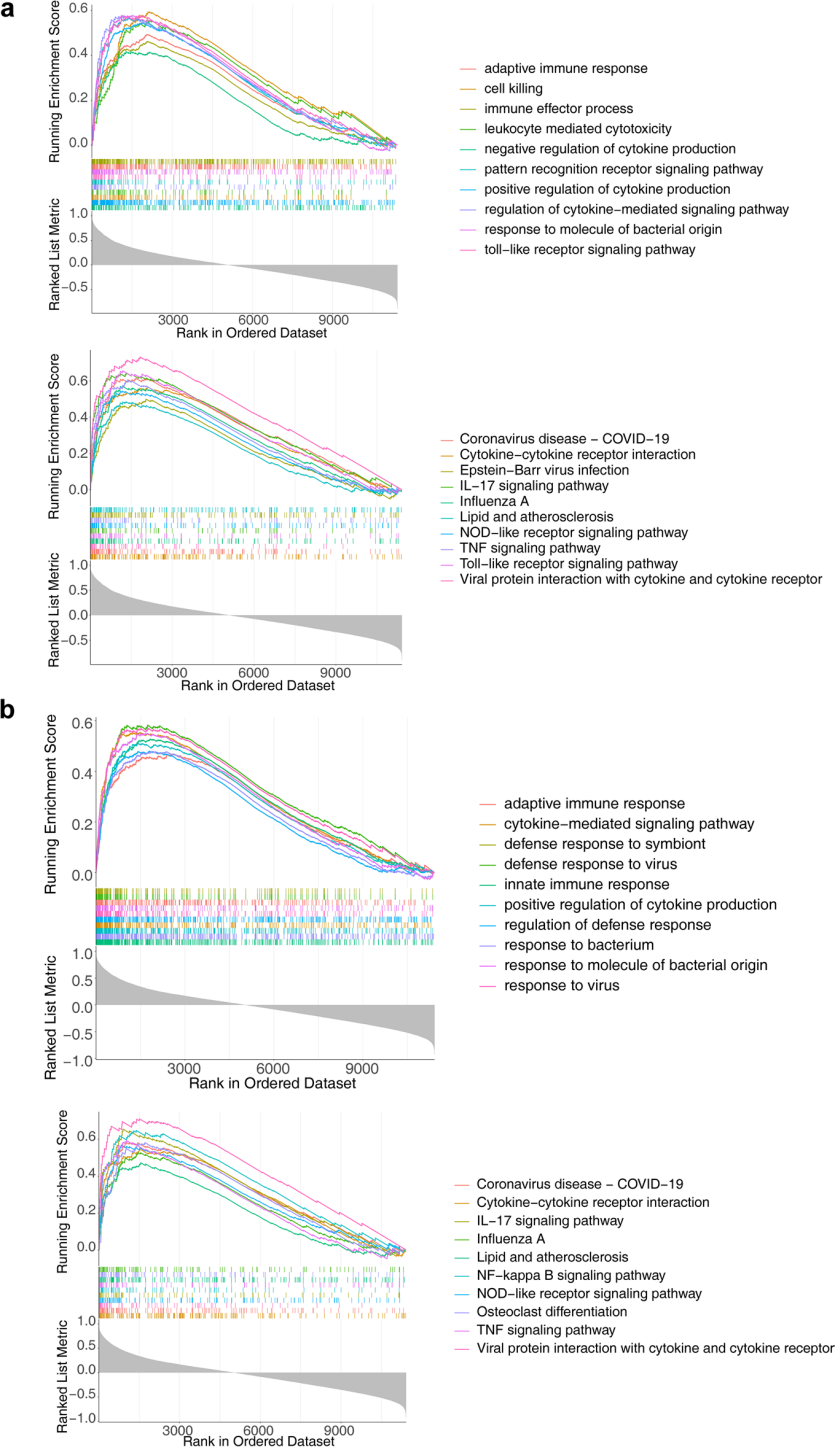
Gene set enrichment analysis (GSEA) of the four biological markers. (**a**) Top10 GO terms and KEGG pathways enriched by Gch1. (**b**) Top10 GO terms and KEGG pathways enriched by Tnfaip3.

GSEA enrichment analysis was performed on the four biological markers in the combined dataset using the default background gene set in the org.Mm.eg.db package, and the significance threshold for ssGSEA was |NES| > 1, p < 0.05, and q < 0.2. The Gch1 was mainly enriched in 1330 GO terms and 115 KEGG pathways (Table S2). As shown in Fig. 6a, Gch1 was associated with toll-like receptor signaling pathway and pathways related to cytokine and immune, such as adaptive immune response, immune effector process, positive/negative regulation of cytokine production and pattern recognition receptor signaling pathway in GO terms. As for KEGG pathways, Gch1 was associated with IL-17 signaling pathway, NOD-like receptor signaling pathway, TNF signaling pathway, and Toll-like receptor signaling pathway (Fig. 6a). Moreover, Tnfaip3 was mainly enriched in 1227 GO terms and 103 KEGG pathways (Table S3). As shown in Fig. 6b, response to virus and bacterium and immune-related pathways (such as adaptive immune response and innate immune response) were enriched in GO terms of Tnfaip3 (Fig. 6b). As for KEGG pathways, Tnfaip3 was associated with IL-17 signaling pathway, NF-kappa B signaling pathway, NOD-like receptor signaling pathway, TNF signaling pathway, and cytokine receptor-related pathways. In conclusion, two biological markers were linked with the occurrence and development of ALI.

### Immune cell infiltration landscape analysis

**Figure 7:**
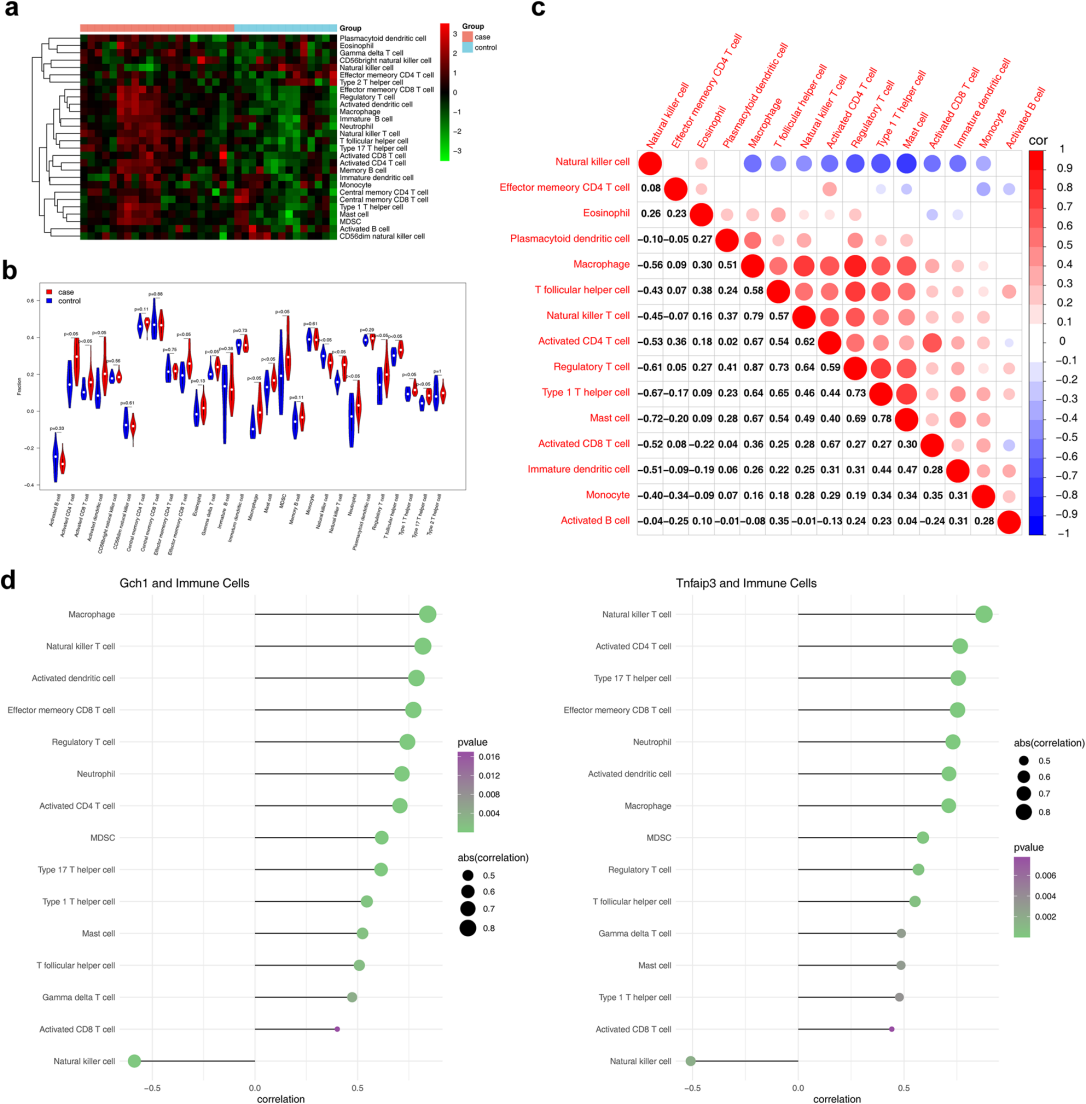
Identification of differential immune cells and analysis of correlation with diagnostic genes. (**a**) The heat map of 28 immune cell infiltration scores. (**b**) The discrepancies of immune cell infiltration between ALI and normal samples. (**c**) The correlation among differential immune cells. Red represents a positive correlation and blue represents a negative correlation. (**d**) The correlation between biological markers and immune cells. The color of the circle represents the direction of the correlation and the size represents the size of the correlation.

The infiltration levels of 28 immune cells in the ssGSEA algorithm were all higher in ALI samples than in control samples (Fig. 7a). A total of 15 types of immune cells were significantly different between the ALI samples and control samples (p < 0.05), including activated CD4 T cell, activated CD8 T cell, activated dendritic cell, effector memory CD8 T cell, gamma delta T cell, macrophage, mast cell, MDSC, natural killer cell, natural killer T cell, neutrophil, regulatory T cell, T follicular helper cell, type 1 T helper cell, and type 17 T helper cell (Fig. 7b). Subsequently, there are correlations among 15 differential immune cells. Among 15 differential immune cells, the mast cell were strongly negatively correlated with natural killer cell, and the regulatory T cell was strongly positively correlated with macrophage (Fig. 7c). Te correlations between Gch1 and the immune cells of macrophage and natural killer T cell were greater than 0.8 (|cor| > 0.8); the correlation between Tnfaip3 and the immune cells of natural killer T cell immune cell was greater than 0.8 (|cor| > 0.8) (Fig. 7d).

### Regulatory mechanism of biological markers and drug prediction

The TF-miRNA-Target network of two biological markers was obtained using miRNet database (https://www.mirnet.ca/) containing 8 TFs, 2 Target genes and 56 miRNAs. The network consisted 66 nodes and 70 relation pairs (Supplementary Fig. 2a). The mmu-let-7c-5p, mmu-mir-124-3p, and mmu-mir-181b-5p were common miRNAs predicted by two biological markers (Supplementary Fig. 2a). Additionally, a Drug-Disease-Target network for the treatment of ALI was constructed, containing 1 disease, 2 target genes, and 3 drugs. The network consisted 6 nodes and 5 relationship pairs (Supplementary Fig. 2b). The GUANINE was predicted by Gch1; the USTEKINUMAB and METHOTREXATE were predicted by Tnfaip3 (Supplementary Fig. 2b).

### Quantitative reverse transcription polymerase chain reaction (qRT-PCR) analysis

**Figure 8:**
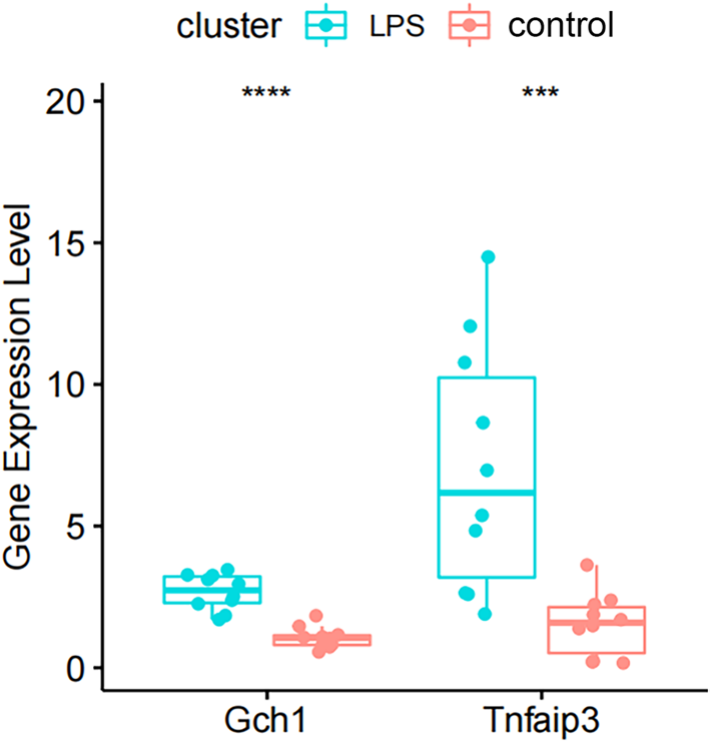
The relative expression of Gch1 and Tnfaip3 in the mice model, ***p value < 0.001, ****p < 0.0001.

In our mice model, the mRNA levels of the two core genes of oxidative stress, including Gch1 and Tnfaip3, were significantly higher in ALI lung tissue than in healthy controls (Fig. 8).

## Discussion

LPS is a specific component of the extracellular membrane of Gram-negative bacteria and is one of the major pathogenic factors causing sepsis and ALI^15^. Mice exhibited a systemic inflammatory response 18 h after intraperitoneal LPS administration^16^. Oxidative stress also plays a key role in the development of ALl and ARDS. Intra-tissue homeostasis requires the maintenance of a complex and delicate balance between oxidants and antioxidants. The disruption of this balance could result in the continued generation of ROS and exceeding the capacity of the antioxidant defense system, which would further lead to the damages of DNA, protein and lipid. This situation could contribute apoptosis^17^, causing pulmonary edema and excessive inflammatory cell infiltration to lung injury^10,18^.

In the present study, 17 core genes were obtained from three independent datasets of differential genes from mice containing ALI and normal mice, and functionally they were associated with the roles of cellular responses to molecules of bacterial origin, cellular responses to biological stimuli and leukocyte migration. They were closely involved in signaling pathways such as IL-17 signaling pathway, TNF signaling pathway and cytokine-cytokine receptor interaction, all of which were associated with inflammatory responses and directly involved in the regulation of ALI. The abnormal expression of these genes and the disruption of their regulated signaling pathways may be related to the development of ALI. Subsequently, we further screened two biomarkers, including Gch1 and Tnfaip3, which were associated with cellular responses to bacterial-derived molecules and lipopolysaccharides and were probably the hub molecules in the pathogenesis of sepsis-associated ALI. The above results were validated by intraperitoneal injection of LPS to establish a mice ALI model, and the mRNA levels of Gch1 and Tnfaip3 were found to be significantly elevated in the lung tissues of ALI mice.

Gch1 is the rate-limiting enzyme for the production of tetrahydrobiopterin (BH4) in the biosynthetic pathway^19^. Higher Gch1 expression contributes to lower levels of oxidative stress^20^. We found that the levels of Gch1 expression were diametrically opposed in different models of cause-induced lung injury. Gch1 expression was decreased in the lungs of mice with hyperoxia-induced lung injury^21^. However, our results showed that Gch1 levels were elevated in the lungs of mice induced by LPS to mimic lung injury caused by bacterial infection compared to healthy controls. This difference may also be related to the difference in Gch1 expression caused by different stimuli. It has been hypothesized that LPS might better stimulate elevated Gch1 responsiveness in oxidative stress. However, this change could also be related to different time periods in which the material was taken for testing. We speculate that a possibility leading to this phenomenon cannot be excluded, that Gch1 is elevated at the beginning of lung injury and subsequently decreased. Whether increasing Gch1 levels within days of lung injury can alleviate lung injury has not been investigated.

TNFAIP3 is an important inhibitor of the pro-inflammatory factor-κB pathway and plays a key role in a variety of diseases. Krusche et al.^22^ performed ex vivo stimulation of Peripheral blood mononuclear cells with LPS and found that activation of the anti-inflammatory process was achieved by increased expression of Tnfaip3. After liver injury, TNFAIP3 exerts dual anti-apoptotic, anti-inflammatory and pro-proliferative effects^23^. Elevated expression of Tnfaip3 can inhibit the progression of inflammation during an inflammatory attack.

In this study, we analyzed the changes in immune cells in ALI and their interrelationships. Among them, mast cells showed a strong negative correlation with NK cells and Treg showed a strong positive correlation with macrophages. Mast cells are engaged in the promotion of inflammatory responses in LPS-induced lung injury and that inhibition of mast cell activation contributes to the suppression of pro-inflammatory gene expression during LPS-induced ALI^24^. During LPS-induced ALI, mice NK cells promote chemokine-mediated neutrophil recruitment and promote inflammation, while depletion of NK cells ameliorates this outcome^25^. After receiving of viral invasion, mast cells secrete cytokines that chemotactic NK cell aggregates and cause them to activate and secrete IFN-γ^26^. This view cannot explain the results of the negative correlation between mast cells and NK cells in this study. Whether the activation of NK cells can form negative feedback on mast cells by inhibiting the production level of mast cells, and the reasons for the negative correlation between the NK cells and mast cells needs further research to explore.

This study revealed correlations between each of the two pivotal genes and immune cells. Gch1 and Tnfaip3 showed the strongest positive correlation with macrophages and NK T cells, respectively. Meanwhile, Gch1 and Tnfaip3 were significantly negatively correlated with NK cells. GCH1 induces immunosuppression of TNBC through metabolic reprogramming and IDO1 upregulation^27^; Xiao^28^ experimentally demonstrated that GCH1 reduces LPS-induced macrophage polarization and inflammation. Furthermore, it has been shown that selective deletion of TNFAIP3 in mice leads to worsening of systemic inflammation and inflammatory dermatoses under homeostatic conditions^29^. Thus, we suggest that GCH1 and TNFAIP3 play a negative regulatory role in the body’s immune system. In summary, we hypothesized that in the LPS-induced ALI model, the abnormally elevated expression of Gch1 and Tnfaip3 negatively regulated NK cells, which disorganized the body’s immune system, and consequently led to the development of the disease.

The medication that acts on Gch1 is probably GUANINE, which has analogs such as VALACYCLOVIR HYDROCHLORIDE. Guanine inhibits the activity of both Gch1 and Gch1 feedback regulatory proteins. However, Gch1 is expected to be increased thus reducing the level of oxidative stress to alleviate the condition in ALI. Therefore, we believe that GUANINE does not alleviate ALI.

Drugs that may act on Tnfaip3 might be USTEKINUMAB and METHOTREXATE (MTX)^30^. The response of psoriasis patients to USTEKINUMAB was associated with Tnfaip3 gene polymorphism^31^, implying that the pharmacotherapeutic effect exerted by USTEKINUMAB might be depend to some extent on the expression of Tnfaip3 or its protein function. Tnfaip3 acts as a negative regulator of nuclear factor-kB, which regulates the inflammatory response of tumor necrosis factor (TNF) by inhibiting the upstream signaling of kB kinase (IKK)^32^. In contrast, MTX^30^ inhibited nuclear factor-kB activation by inhibiting IKK and did not reveal the possibility of altering Tnfaip3 expression or protein function, but may contribute to mitigate the effects of ALI.

This research was the first time to systematically investigate the role of oxidative stress-related genes in ALI and perform immune infiltration-related analysis by bioinformatics technology based on the data in the GEO database. In addition, we also performed preliminary validation of our findings by constructing an animal model. However, there were also shortcomings in this study. First, our analysis was develop based on a limited sample of public databases, and expanding the sample size was an urgent issue. Although we obtained biological markers related to oxidative stress for ALI screening, their roles and mechanisms needed to be further investigated and validated. In addition, we had predicted potential drugs based on biomarkers, their effectiveness needed to be validated in the clinic.

## Conclusion

In conclusion, 2 OSRGs (Gch1 and Tnfaip3) with higher expression in ALI samples than in control samples were identified as biological marker for LPS-induced ALI, and they may be involved in the immune-related pathways. In addition, 15 differential immune cells might play an important role in the development and progression of ALI, especially macrophage, natural killer T cell, and natural killer cell. USTEKINUMAB, MTX, and GUANINE may be potential therapeutic agents to alleviate ALI. Thus, we believe that our findings will provide a new theoretical basis for further research on the role of oxidative stress in ALI, and will also provide new targets for the diagnosis and treatment of ALI. We will continue to focus on the role of Gch1 and Tnfaip3 in ALI and further explore their mechanisms of action.

## Materials and methods

### Data source

The GSE102016, GSE104214, GSE16409, GSE17355, and GSE18341 datasets were acquired from the GEO database (https://www.ncbi.nlm.nih.gov/geo/). Among these datasets, the untreated samples from the control group and wild-type samples treated with LPS were selected for subsequent analysis, and the data information was shown in Table S4. Additionally, 1399 OSRGs were downloaded from the GeneCards database (https://www.genecards.org) with Relevance score ≥ 7, and then transformed these human genes to obtain 1292 mice homologous genes. The GSE16409, GSE18341 and GSE102016 datasets were background corrected, quantile normalized and merged as a combined dataset, then batch effects were removed using the SVA package (version 3.42.0)^33^.

### Screening of ALIRGs

The R package WGCNA (version 1.7-3)^34^ was used to construct a co-expression network in the combined dataset, and the ALI samples and control samples were used as the trait data of WGCNA to search for ALIRGs. Firstly, the samples were clustered and outlier samples were removed to ensure the accuracy of the further analysis. Then a sample cluster and the heatmap of clinical traits were constructed. The soft threshold of the data determined to ensure that the interaction between genes conformed to the scale-free distribution to the greatest extent. The phylogenetic clustering tree among genes was obtained on the basis of the adjacency relationship and the similarity between genes. The minimum number of genes in each gene module was set to 200 according to the criteria of the hybrid dynamic tree cutting algorithm. Subsequently, modules with the highest disease relevance and key ALIRGs they contained were selected.

### Screening of DE-ALI-OSRGs

The limma package (version 3.50.0)^35^ was used to compare the differences in gene expression between the ALI samples and control samples in the combined dataset. The ggplot2 package (version 3.3.5)^36^ was used to draw volcano plots to show the DEGs. The VennDiagram (version 1.7.1)^37^ was used to obtain the DE-ALI-OSRGs, that was the intersection of the ALIRGs, DEGs and OSRGs. In addition, the correlations between intersecting genes were calculated.

### Enrichment analysis

The clusterProfiler package (version 4.2.2)^38^ was used to implement the GO and the Kyoto Encyclopedia of KEGG enrichment of DE-ALI-OSRGs, and the enrichment results were visualized using GOplot (version 1.0.2)^39^ and enrichplot packages (version 1.10.2)^40^.

### Screening of biological markers

The LASSO and SVM-RFE machine learning models were constructed in the combined dataset to screen candidate biological markers in the ALI based on the DE-ALI-OSRGs. The LASSO algorithm was implemented by “glmnet” package^41^ (version 4.0-2) with parameters set to famil = binomial and type.measure = class. The e1071 package^42^ (version 1.7-9) was employed to implement SVM-RFE algorithm. The overlapping genes were selected from the results of the LASSO and SVM-RFE, and these genes were considered as candidate biological markers.

In the combined dataset, GSE104214 dataset and GSE17355 dataset, the wilcox.test was performed to verify the differential expression of biological markers between the ALI samples and control samples, and scatter points graphs were visualized to verify the expression levels of the candidate biological markers by the ggpubr package (version 0.40)^43^.

To explore the prediction of candidate biological markers on sample traits and select the biological markers, ROC analysis was performed on the combined dataset, GSE104214 dataset, and GSE17355 datasets for candidate biological markers using the pROC package^44^ (version 1.18.0). The genes with differential expression in both datasets and area under the curve (AUC) values greater than 0.9 were treated as biological markers. Finally, the STRING (https://string-db.org) website was used to construct a PPI network with the confidence = 0.4 to explore the interaction among biological markers. The PPI network was drawn by Cytoscape (version 3.8.2)^45^ software.

### GSEA enrichment analysis

The clusterProfiler (version 4.2.2)^38^ was applied to perform the GSEA enrichment analysis to find the functions and pathways of the biological markers. GSEA enrichment analysis was performed based on the default background gene set in the org.Mm.eg.db package.

### Immune cell infiltration landscape analysis

In order to study the difference of immune infiltration between the ALI samples and control samples, ssGSEA was used to estimate the infiltration of immune cell types in the gsva package (version 1.42.0)^46^. The wilcox.test was used to compare the differences between different immune cells in the combined dataset between the ALI samples and control samples, and the violin plot was drawn using the vioplot package (version 0.3.7) to visualize the comparison results. Pearson correlation analysis was performed on the different immune cells using the R language and plotted correlation using the corrplot package (version 1.0.12)^47^. In addition, the Pearson correlation coefficient between biological markers and immune cells based on the combined dataset were delved.

### Regulatory mechanism of diagnostic genes and drug prediction

The transcriptional regulatory network of TF-miRNA-Target was predicted by biological markers in the miRNet database (https://www.mirnet.ca/). The drug-gene interaction database (DGIdb; https://dgidb.genome.wustl.edu/) identified biological markers for therapeutic drugs. The networks mentioned above were drawn by Cytoscape software (version 3.8.2)^45^.

### Animal experiments

#### Mice

The Mice (C57BL/6 mice, male, aged 8–10 weeks) of this study were purchased from the Hunan Shrek Jingda Experimental Animal Co., Ltd. (Changsha, China).

#### LPS/ALI model

To establish an LPS-induced ALI model, 20 C57BL/6 mice were divided equally into 2 groups, 10 of which were injected intraperitoneally with a single dose of PBS premixed with LPS (10 mg/kg, Sigma Aldrich, Cedex, France) while the other 10 with PBS liquid^48^. LPS-induced ALI mice were molded for 20 h, 1% pentobarbital (50 mg/kg) was injected intraperitoneally for anesthesia, then the eyes were removed and bled, the mice were sacrificed after cervical dislocation, and lung tissue was taken for real-time quantitative PCR analysis. The studies involving animal experiments were reviewed and approved by the Animal Care and Use Committee of Central South University (CSU-2023-0080), all methods were carried out in accordance with relevant guidelines and regulations. All procedures performed in this study involving animal experiments were in accordance with the ARRIVE guidelines (Animal Research: Reporting of In Vivo Experiments)^49^. Moreover, the execution of animals was according to the American Veterinary Medical Association (AVMA) Guidelines for the Euthanasia of Animals (2020).

#### Validation of the biological markers by qRT-PCR

Mice lung tissue were treated with TRIzol reagent (50 mg tissue/1 mL, Invitrogen, Carlsbad, CA) at room temperature for 5 min, then centrifuged at 4000×*g* for 15 min at 4 °C with chloroform. The upper aqueous phase was aspirated into a new EP tube, treated with isopropyl alcohol for 10 min, and centrifuged at 4000×*g* for 10 min (both at 4 °C). The precipitate was washed with 75% ethanol, and the RNA was solubilized by adding RNase-free DEPC and stored at − 80 °C. After the detection of RNA purity and concentration using the spectrophotometer (Thermo Fisher, Waltham, MA, USA), TransScript® Reverse Transcriptase [M-MLV, RNaseH-] (AT101-02, Transgen, Beijing, China) was used for the reverse transcription. The qPCR reactions were then performed on an ABI 7900HT Fast platform using the TransStart® Green qPCR SuperMix (AQ101-01, TransGen, Beijing, China) according to the manufacturer’s instructions^50^. The sequences of the forward and reverse primers were displayed in Table 1 (Tsingke Biotech, Beijing, China). The relative expression of each biological markers was determined by the 2^−ΔΔCt^ method with GAPDH as the internal reference.

**Table 1:** The sequences of primers for biological markers.

Primer	Sequence
Mice GAPDH forward	5ʹ-ACGGCACAGTCAAGGCAGA-3ʹ
Mice GAPDH reverse	5ʹ-GTGATGGCGTGGACAGTGG-3ʹ
Mice Gch1 forward	5ʹ-GTCCTTGGTCTCAGTAAACTTGCCAGG-3ʹ
Mice Gch1 reverse	5ʹ-GCCCAGCCAAGGATAGATGCAG-3ʹ
Mice Tnfaip3 forward	5ʹ-TCAACTGGTGTCGAGAAGTCC-3ʹ
Mice Tnfaip3 reverse	5ʹ-CAAGTCTGTGTCCTGAACGC-3ʹ

#### Statistical analysis

All analyses were conducted using R language (https://www.r-project.org/). The wilcox.test was utilized to evaluate the differential expression levels of biological markers between the ALI samples and control samples. All experimental data was expressed as mean ± standard deviation. Comparisons between the two groups were implemented using the Student’s t-test. If not specified above, *p* < 0.05 was regarded as statistically significant.

### Ethics statement

The GEO database is a public dataset. The animals involved in the database have been approved by ethics. Users can download the relevant data for free to conduct research and publish related articles. Our research is based on open-source data. The animal experiments in this study followed the ethical requirements of the Animal Care and Use Committee of Central South University (No. 2017sydw00284), the ARRIVE guidelines (Animal Research: Reporting of In Vivo Experiments) and the American Veterinary Medical Association (AVMA) Guidelines for the Euthanasia of Animals (2020).

### Supplementary Information

Supplementary Figures.

Supplementary Table S1.

Supplementary Table S2.

Supplementary Table S3.

Supplementary Table S4.

## Data Availability

The datasets generated and analyzed during the present study are available in the Gene Expression Omnibus (GEO, https://www.ncbi.nlm.nih.gov/geo/) database (Accession Number: GSE102016, GSE104214, GSE16409, GSE17355 and GSE18341)/Supplementary Material.

## Abbreviations

ALI: Acute lung injury
OSRGs: Oxidative stress-related genes
ALIRGs: ALI-related genes
DEGs: Differentially expressed genes
DE-ALI-OSRGs: Differentially expressed OSRGs in ALI
PPI: Protein–protein interactions
SVM-RFE: Support vector machine recursive feature elimination
ARDS: Acute respiratory distress syndrome
ROS: Reactive oxygen species
LPS: Lipopolysaccharide
GO: Gene Ontology
KEGG: Kyoto Encyclopedia of Genes and Genomes
GSEA: Gene set enrichment analysis
