# Supplementary material for: Exploration of the role of oxidative stress-related genes in LPS-induced acute lung injury via bioinformatics and experimental studies
Source: Sci Rep. 2023 Dec 9;13:21804. doi: 10.1038/s41598-023-49165-3 (PMC10710410; doi:10.1038/s41598-023-49165-3)
Supplement: Supplementary file 1 — Supplementary Figures. [file 41598_2023_49165_MOESM1_ESM.docx]

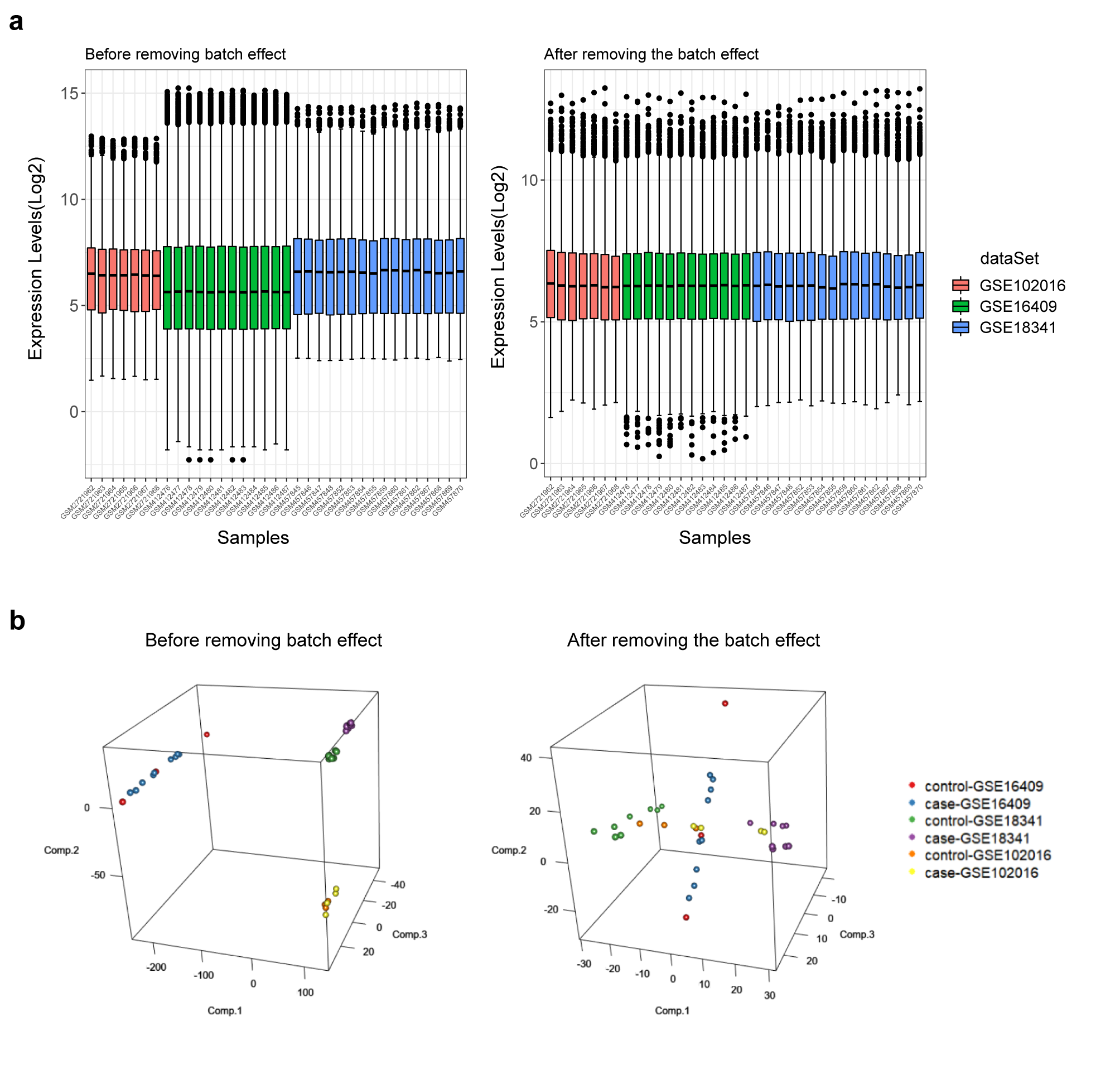
**Supplementary Figure 1:** Batch effects removal for the combined dataset. (a). The expression levels of each sample before and after batch calibration for GSE16409, GSE18341 and GSE102016 data sets. (b). PCA result before and after batch calibration.


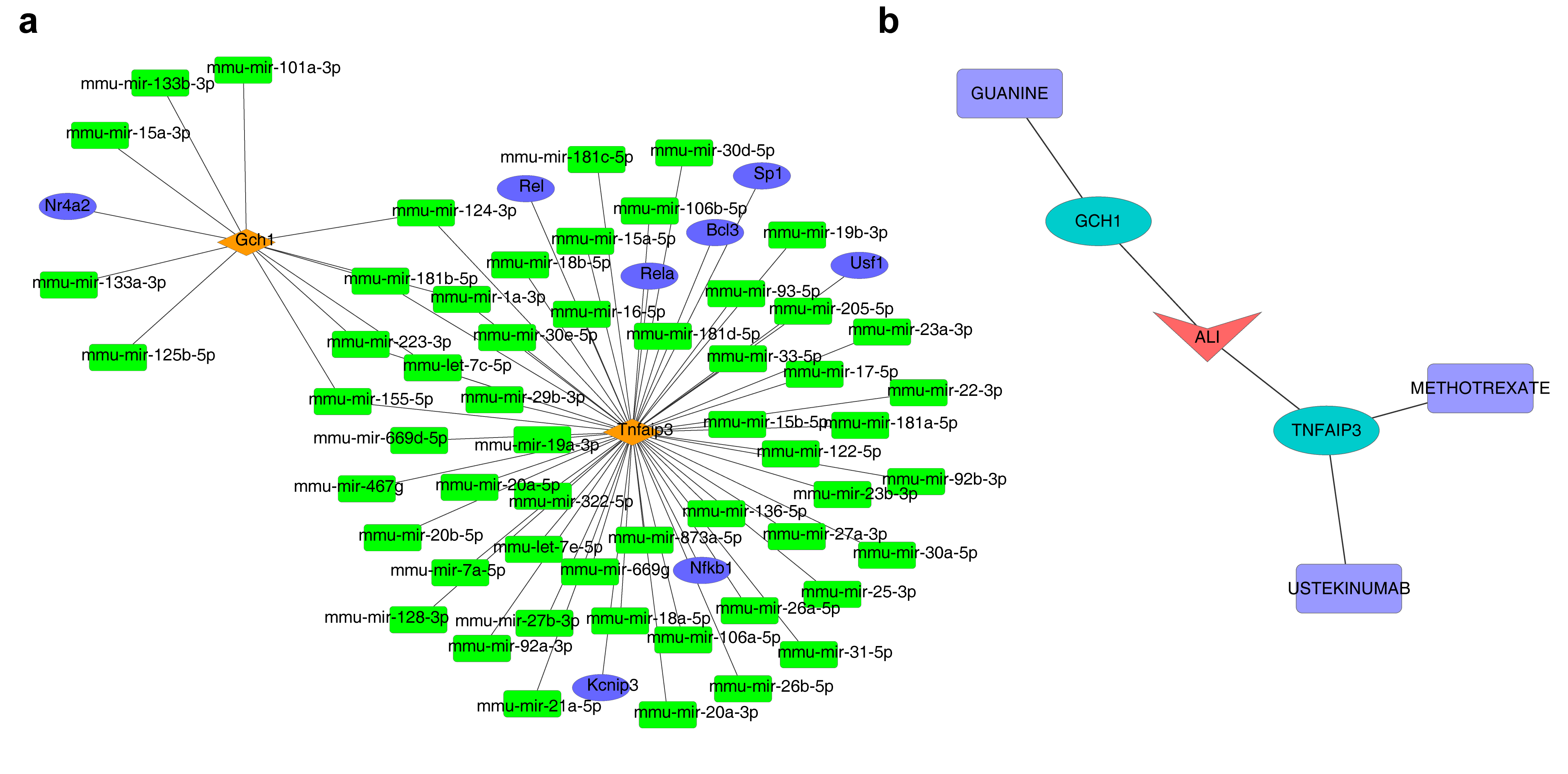


**Supplementary Figure 2:** The regulatory network for the the biological markers. (a). The TF-miRNA-target gene transcriptional regulatory network. Purple circle represents TF, green box represents miRNA, and orange diamond represents target gene. (b). The Drug-Disease-Target network. Purple rectangles represent drugs, blue-green ovals represent target genes, and red triangles represent diseases
